# A Multibody Model for Predicting Spatial Distribution of Human Brain Deformation Following Impact Loading

**DOI:** 10.1115/1.4046866

**Published:** 2020-05-15

**Authors:** David Gabrieli, Nicholas F. Vigilante, Rich Scheinfeld, Jared A. Rifkin, Samantha N. Schumm, Taotao Wu, Lee F. Gabler, Matthew B. Panzer, David F. Meaney

**Affiliations:** Department of Bioengineering, University of Pennsylvania, 240 Skirkanich Hall, 210. S. 33rd Street, Philadelphia, PA 19104; Department of Mechanical and Aerospace Engineering, Center for Applied Biomechanics, University of Virginia, P.O. Box 400237, Charlottesville, VA 22904; Departments of Mechanical and Aerospace Engineering and Biomedical Engineering, Center for Applied Biomechanics, University of Virginia, P.O. Box 400237, Charlottesville, VA 22904; Departments of Bioengineering and Neurosurgery, University of Pennsylvania, 240 Skirkanich Hall, 210 S. 33rd Street, Philadelphia, PA 19104

## Abstract

With an increasing focus on long-term consequences of concussive brain injuries, there is a new emphasis on developing tools that can accurately predict the mechanical response of the brain to impact loading. Although finite element models (FEM) estimate the brain response under dynamic loading, these models are not capable of delivering rapid (∼seconds) estimates of the brain's mechanical response. In this study, we develop a multibody spring-mass-damper model that estimates the regional motion of the brain to rotational accelerations delivered either about one anatomic axis or across three orthogonal axes simultaneously. In total, we estimated the deformation across 120 locations within a 50th percentile human brain. We found the multibody model (MBM) correlated, but did not precisely predict, the computed finite element response (average relative error: 18.4 ± 13.1%). We used machine learning (ML) to combine the prediction from the MBM and the loading kinematics (peak rotational acceleration, peak rotational velocity) and significantly reduced the discrepancy between the MBM and FEM (average relative error: 9.8 ± 7.7%). Using an independent sports injury testing set, we found the hybrid ML model also correlated well with predictions from a FEM (average relative error: 16.4 ± 10.2%). Finally, we used this hybrid MBM-ML approach to predict strains appearing in different locations throughout the brain, with average relative error estimates ranging from 8.6% to 25.2% for complex, multi-axial acceleration loading. Together, these results show a rapid and reasonably accurate method for predicting the mechanical response of the brain for single and multiplanar inputs, and provide a new tool for quickly assessing the consequences of impact loading throughout the brain.

## Introduction

In the past five years, the prevalence of mild traumatic brain injury (mTBI) has increased significantly from both widespread changes in monitoring athletes during competition and increased awareness of mTBI symptoms and diagnosis. Since 2006, the estimated concussions occurring annually has grown from 1.7–2 million to 2.8 million in 2013 [1,2], with over 2.5 million self-reported concussions occurring in high-school athletes alone in 2017 [3]. Although most mTBI have no long-lasting neurological impairments on their own, a subset of concussions can lead to prolonged deficits, especially in persons with repeated mTBIs [4–7]. From both acute and prolonged care, the aggregate cost of mTBI is now estimated to exceed 70 billion U.S. dollars annually [8].

It is well known that concussion can occur from the rotational motion experienced by the head during direct or indirect head impact [9]. Due to the soft material properties of the brain (see reviews: [10–12]), these rotational motions cause substantial deformations throughout the gray and white matter [13–15]. In turn, these intracranial strains can cause both structural and functional impairment of brain tissues [16,17]. A key feature of understanding and, eventually, reducing concussion risk is to determine more exact relationships between the external kinematic loading applied to the head and the subsequent deformation of the intracranial contents. Simple spring-mass-damper models of the brain have characterized the impact response, natural frequency, and the surrogate strains experienced by the brain prior to injury [18–22]. A generation of analytical models provided more spatial estimates of the brain but were limited to simple geometries (summarized in Ref. [23]). With their ability to simulate complex geometries and loading inputs, finite element approaches quickly eclipsed both of these approaches to become the most common current methods relating external mechanical loading to the potential areas of brain injury.

A series of computational models can be used to study how the brain deformation response to impact is influenced by brain size, structure, and physical properties. Finite element (FE) models are the most commonly used tool and, although they offer significant insight into injury mechanisms, FE simulations can be computationally expensive and require hours to simulate impact events lasting less than 100 ms. In many studies, the computational cost is offset by the significant benefit provided by the ability to pinpoint areas of vascular injury [24–26], the relative fraction of brain volume damaged [27–29], or even the estimated changes in brain networks from a given impact [30].

An alternative method for achieving an estimate of stress/strain throughout the brain is the material point method, which does not suffer from some of the drawbacks commonly associated with FE models [31,32]. These FE limitations include the possibility of significant mesh warping during the simulation, the difficulty of modeling nearly incompressible materials, and limited material models to simulate the nonlinear, viscoelastic behavior of brain tissue. However, neither the finite element nor the material point model is well designed for rapidly assessing, i.e., within seconds—whether an impact poses any risk for brain injury. Rapid injury risk analysis would be particularly helpful in the headgear design environment, where the impact of design changes could be executed quickly and facilitate an iterative process that would yield a prototype helmet design more rapidly than a design that requires finite element modeling. In addition, rapid injury risk calculations would also assist with the interpretation of sensor data recording head acceleration exposures in the field of play, significantly improving the ability to detect players who need to be evaluated for possible symptoms of mTBI.

Recent efforts to develop a single degree-of-freedom model of the brain in response to a rotational motion produced a tool that successfully approximated the peak brain deformation to a three-dimensional (3D) acceleration input [33,34]. In this paper, we extend this approach and develop a multibody-based tool, where we estimate deformations throughout the brain during an impact event. We use this model to estimate the brain motions that occur across an anatomic plane and extend this analysis to predict deformations that occur throughout the brain from simple and more complex loading. Across a range of mechanical exposure conditions, we find that combining machine learning (ML) techniques with the MBM predictions provides a fast and reasonably accurate estimate of tissue deformations calculated using a finite element model (FEM) of the head. Together, these results demonstrate the potential for quickly computing the brain deformation response to impact. In a larger scope, this approach provides the opportunity to more rapidly identify mechanical exposures that could lead to traumatic brain injury.

## Materials and Methods

### Development of Planar Multibody Models.

**Fig. 1:**
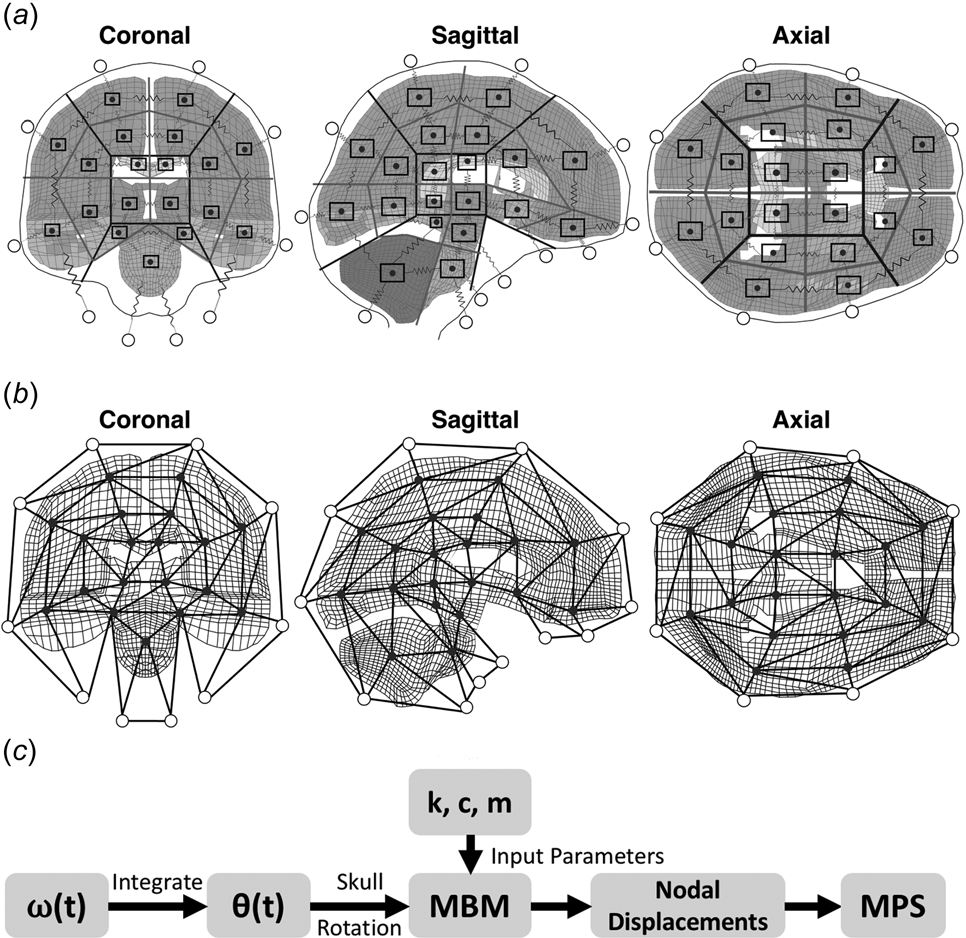
Creation of planar MBMs: (*a*) Human full brain FEM mesh with overlay of mass-spring-damper system from MBMs; (*b*) nodes were connected into triangular elements and used for calculating true strain; and (*c*) flow diagram to calculate maximum principal strains from MBM inputs

Planar multibody models (MBM) were implemented in simscape (version 4.2, The Mathworks, Natick, MA) as a coupled mass-spring-damper system. To develop the human model structure, the Global Human Body Models Consortium (GHBMC) owned 50th percentile male FEM was partitioned along the midline in the coronal, sagittal, and axial planes to create 19–20 coarse elements in each plane. MBM nodes were placed at the center of each coarse element (Fig. 1(*a*)), with each point mass corresponding to a brain node in the FEM. Masses for each brain node in the MBM reflected the proportional area covered by each coarse element in the planar model. Additional MBM nodes were placed at the locations of known FEM skull elements, and these additional nodes were used to deliver a prescribed rotational motion to the model. Springs and dampers connected each brain node to surrounding nodes, while skull nodes connected to the closest brain node (Fig. 1(*b*)). For a given point mass, each spring was assumed to act through the center of mass and yielded a force on the point mass

(1) $$F=K_{1}\delta_{1\underline{e_{1}}}$$

where F is the force acting along the spring in the direction specified by $e_{1}$, and $K_{1}$ and $\delta_{1}$ are the spring constant and displacement of the spring, respectively. Across all four springs acting on a point mass, the net elastic force on the point mass was the sum of the individual spring elements (*K*_i_) in the direction of their respective unit vectors (**e**_i_)

(2) $$\underline{F_{\mathrm{elastic}}=K_{1}\delta_{1}\underline{e_{1}}+K_{2}\delta_{2}\underline{e_{2}}+K_{3}\delta_{3}e_{3}+K_{4}\delta_{4}\underline{e_{4}}}$$

which can be represented in matrix form

(3) [Felastic,xFelastic,y]=[K1e1x¯K2e2x¯K1e1y¯K2e2y¯ K3e3x¯K4e4x¯K3e3y¯K4e4y¯][δ1δ2δ3δ4]

We implemented damping proportional to *K* using a damping factor (*β*) and the corresponding displacement rates ($\dot{\delta })$

(4) $$F_{\mathrm{damping}}=\beta K\dot{\delta },$$

or

(5) [Fdamping,xFdamping,y]=β[K1e1x¯K2e2x¯K1e1y¯K2e2y¯ K3e3x¯K4e4x¯K3e3y¯K4e4y¯][δ˙1δ˙2δ˙3δ˙4]

and combined these to develop the governing equations of motion

(6) $$\left[\begin{array}{c} Ma_{x}\\ Ma_{y} \end{array}\right]=\left[\begin{array}{c} F_{\mathrm{elastic},x}\\ F_{\mathrm{elastic},y} \end{array}\right]+\left[\begin{array}{c} F_{\mathrm{damping},x}\\ F_{\mathrm{damping},y} \end{array}\right]$$

Skull nodes were driven using position-time histories of either simple or more complex loading pulses (described below) (Fig. 1(*c*)). MBM simulations were solved using a Dormand–Prince method [35] based ordinary differential equation solver.

### Three-Dimensional Finite Element Simulations of Nodal Displacement in Response to Rapid Planar Rotation.

Idealized sinusoidal rotational motions were applied to the human MBM and the GHBMC FEM to validate the model and evaluate predictive capability, as in Ref. [34]. Briefly, angular velocities and accelerations from 660 sled, crash, and pendulum tests were analyzed, and a single sinusoidal acceleration pulse was developed across the range of impact pulses. Angular accelerations and velocities ranged from 0.1 to 15 krad/s^2^ and 1–100 rad/s, respectively. For each kinematic variable, 17 values across the range were selected. The maximum principal strain (MPS) and nodal position time history were recorded for each simulation and later compared to results from the MBM. Impact times were limited to avoid erroneous portions of the kinematic parameter space (*n* = 75 of 280 total simulations), yielding 205 FEM simulations per anatomic plane (< 60 ms; [15]).

### Helmet Impact Testing to Estimate Complex Three-Dimensional Head Motions.

Six-degree-of-freedom (DOF) head kinematics from laboratory tests involving a helmeted dummy head-neck were used to estimate complex loadings that may occur during a helmet-to-helmet impact in American football. Laboratory tests were obtained from a larger study involving impacts to various helmets at multiple speeds and locations [36]. MPS for these impacts were previously obtained from FEM simulations using the GHBMC [33]. A total of 96 impacts involving four different helmets, eight locations, and three speeds (5.5, 7.4, and 9.3 m/s) were collected from the previous studies and used in this study for testing of MBM performance.

### Anthropomorphic Test Dummy Reconstructions of On-Field Head Impacts for Evaluation of Model Fidelity.

We used a set of video-based reconstructions for striking and struck players in professional football [37] to further compare our MBM results with FE simulations. Initially based on anthropomorphic test dummy reconstructions of 31 impact events, these kinematic loading conditions were reexamined in a recent report [38] and updated to provide more accurate 3D kinematic loading conditions for 53 specific impact scenarios that encompassed helmet-to-helmet impacts. We utilized the estimated 3DOF rotational velocity inputs for both the hybrid machine learning-MBM and the FEM, truncating simulation times to avoid erroneous portions of the kinematic loading profile (<60 ms, [38]).

### Validation and Optimization of Planar Multibody Model.

**Fig. 2:**
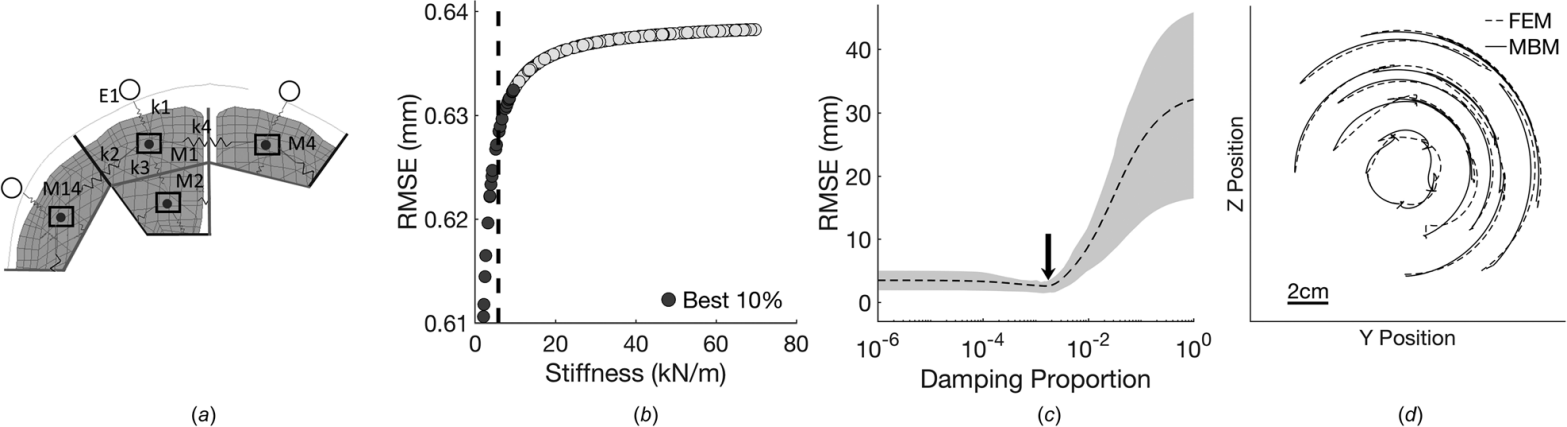
Overview of optimization process for MBMs: (*a*) planar MBMs were split into subdomains for spring stiffness optimization and (*b*) a range of spring stiffnesses for each subdomain was tested. The mean stiffness of the 10% of cases (dashed line) with the minimum RMSE was implemented for the subdomain. Springs existing in multiple subdomains were assigned the mean stiffness from subdomain optimization. (*c*) Planar MBMs were tested for an optimal range of proportional damping values for all springs, with 0.15% damping (arrow) used for all springs in all planes. Shading represents 25th to 75th percentiles. (*d*) Representative plot comparing nodal position histories of the MBM and FEM in the coronal plane.

To optimize the stiffnesses and damping factors of all springs in each multibody model, we divided a planar model into smaller subdomains (Fig. 2(*a*)). For each subdomain, positions of the adjacent nodes were prescribed to match the corresponding node from the FEM simulation. The stiffnesses of the springs connected to the central node in the subdomain were varied over a range of 2000–70,000 N/m (*n* = 250 simulations total per subdomain). For a given haversine acceleration pulse, the position history of the central node was compared to the corresponding position history of the equivalent node in the FEM, and the resulting root-mean-squared-error (RMSE) of position was computed for each simulation. The simulations leading to the smallest 10% of RMSEs over all simulations were selected, and the spring stiffnesses used in this simulation subset were averaged to identify the optimal stiffness for each subdomain. For springs shared between two subdomains, the optimal stiffnesses were averaged from the values derived from each subdomain analysis. To achieve a robust set of stiffness values that would apply over a broad kinematic loading, we determined the optimal stiffness values for 15 different kinematic loading conditions that spanned the peak changes in angular velocity (10–50 rad/s) and peak angular accelerations (0.5–7.9 krad/s^2^) that occur in helmet impact tests. The resulting stiffness value for each spring in the MBM was averaged from the values obtained from these 15 loading simulations.

Following spring stiffness optimization, each full planar model was run over a range of damping factors, from 0% to 1%. We compared the RMSE at different damping factors, determining which damping factors yielded results that were not different from each other. With this subgroup of damping factors, we selected a single damping factor and kept it constant across the models. Resulting models were then compared to the FEM to ensure nodal position accuracy.

### Comparison of Maximum Principal Strain Between Finite Element and Multibody Model.

To evaluate the ability of the MBM to accurately predict the strain calculated from a 3D FEM, we computed the Hencky (true) strain tensor components for all triangular elements that connected triads of adjacent nodes in the MBM. Using three points in the undeformed (*a*_1_, *a*_2_, *a*_3_) and deformed (*x*_1_, *x*_2_, *x*_3_) state for each triangle, we computed the lengths of the triangle sides in both states (ds,${ds}_{o}$) and use this to calculate Green strain ($E^{G}$)

(7) $${ds}^{2}-{{ds}_{o}}^{2}=2E_{ij}^{G}{da}_{i}{da}_{j}$$

from which we computed Hencky strain ($E^{H}$)

(8) $$E^{H}=\frac{1}{2}ln(I+2E^{G})$$

where **I** is the identity matrix. The Hencky strain matrix was used to compute principal strains for each element in the MBM. The MPS was determined as the larger of the two principal strains in that element. The 95th percentile MPS, a common metric for estimating brain injury risk [39], for a MBM was selected from the list of MPS values from each triangular element in the MBM for a given input acceleration pulse.

### Development of Machine Learning-Assisted Multibody Model Tool.

Once we identified optimal stiffness and damping values to approximate the finite element response for each planar MBM, we used ML techniques to improve the correlation between the MBMs and the corresponding FEM. We created a regression model in each plane, composed of an ensemble of 30 regression trees trained with the LSBoost algorithm [40]. We used the 95th percentile MPS computed from the MBM, the peak angular velocity, and the peak angular acceleration as features in the ML model to predict the 95th percentile MPS in the FEM. To determine if ML could predict MPS across the entire parameter space, all 280 sinusoidal impact traces were utilized, including those left out of MBM-only analysis. Of the 280 traces in each plane, 60% (*n* = 168, selected randomly) were used for training and validation. Models were validated with fivefold cross validation. Model testing was conducted on the remaining 40% (*n* = 112) of the sinusoidal traces to analyze its predictive capability. Models were labeled according to the dataset used to train them, e.g., “MBM-ML-sinusoid” refers to ML models trained using haversine acceleration pulses.

To extend the model for predicting the MPS that occurred when rotational motion occurred simultaneously across three planes, the resultant of the maximum principal strain (MPS_res_) in each plane (MPS_x_, MPS_y_, MPS_z_)

(9) $${\mathrm{MPS}}_{\mathrm{res}}=\sqrt{\left({\mathrm{MPS}}_{x}^{2}+{\mathrm{MPS}}_{y}^{2}+{\mathrm{MPS}}_{z}^{2}\right)}$$

was taken and compared with the MPS from the FEM, consistent with the previous work [33]. To determine the relative improvement provided by ML-assisted predictions of the brain deformation compared to the MBM alone, we compared resultant errors of each approach to the calculation of the FEM. For this comparison, we computed the relative error

(10) $$\mathrm{Relative}\;\mathrm{error}\;(\%)=\frac{\left|{\mathrm{MPS}}_{\mathrm{MBM}}-{\mathrm{MPS}}_{\mathrm{FEM}}\right|}{{\mathrm{MPS}}_{\mathrm{FEM}}}\times 100$$

where MPS_MBM_ and MPS_FEM_ are the MPS (95th percentile) from the MBM and FEM, respectively. Until this point, our ML was restricted to predicting the mechanical response from simple haversine acceleration pulses. Helmet impacts typically contain acceleration components along three axes and may contain more than one phase of acceleration. We next used two approaches to evaluate if ML-assisted MBMs would be well suited for these more complex acceleration pulses. Our first approach used the optimized, ML-assisted models for each plane (see above MBM-ML-sinusoid models), applied the corresponding planar kinematic inputs to each model, and then estimated the MPS for the complex pulse as the resultant of the MPS from each ML-assisted planar MBM. Our second approach relied only on the loading conditions from the 96 complex professional football helmet impact cases to create a set of new models (MBM-ML-helmet) that were separately trained and validated using only these helmet impacts. The advantage of this second approach was creating a model optimized for actual impact conditions, rather than possibly losing accuracy by fitting the model to a broader range of loading conditions that extend well beyond typical impact conditions. Approximately, 60% (*n* = 57) of the professional football helmet impact cases were used to train and validate the models. Models ranged from having three (e.g., MBM MPS in each plane) to nine features (all three MPS parameters, all six kinematic parameters in each plane). All models were created with an ensemble of 30 regression trees trained with the LSBoost algorithm and validated with fivefold cross validation. Model performance was evaluated using three metrics: RMSE, *R*^2^, and mean absolute error (MAE). The remaining 40% (*n* = 39) of complex cases were reserved for testing the best performing model from the training and validation phases.

### Generation of Machine Learning-Based Regional Maximum Principal Strain Predictions.

Given that it is likely that injury risk prediction will be influenced by where the peak brain deformation occurs during an impact exposure, we next created a set of ML models for each triangular element in the planar MBMs to predict the corresponding peak FE MPS in the same location. To avoid possible errors from individual element variations, we selected a group of FEs that captured 10% of the total triangular area of each MBM triangle, averaging the MPS from these elements to develop the output to the regression model. ML models were not created for triangular MBM elements which (1) did not have any FEM elements within the calculated radius or (2) had a centroid in nonbrain matter (e.g., a ventricle or cerebrospinal fluid). From a possible total of 137 element models, we created 120 element-specific ML models. The MBM- and kinematics-based features of the element-specific ML models were identical to those in the model used to predict whole brain MPS.

## Results

### Optimization of Multibody Model Parameters.

Across a range of brain stiffness values and model subdomains, we observed the residual error in displacements predicted from the subdomain central node and the closest FEM node (Fig. 2(*b*)). RMSE dependence on spring stiffness was variable depending on the associated subdomain. Stiffnesses did not correlate with nodal location or boundary proximity. We also found that the error residuals for each planar multibody model were influenced by the proportional damping specified for the model (Fig. 2(*c*)), with 0.15% as the optimal proportional damping factor. Optimization in each plane resulted in a close match of nodal trajectories to the motion of equivalent nodes in a FEM subjected to the same rotational input pulses (Fig. 2(*d*)).

**Fig. 3:**
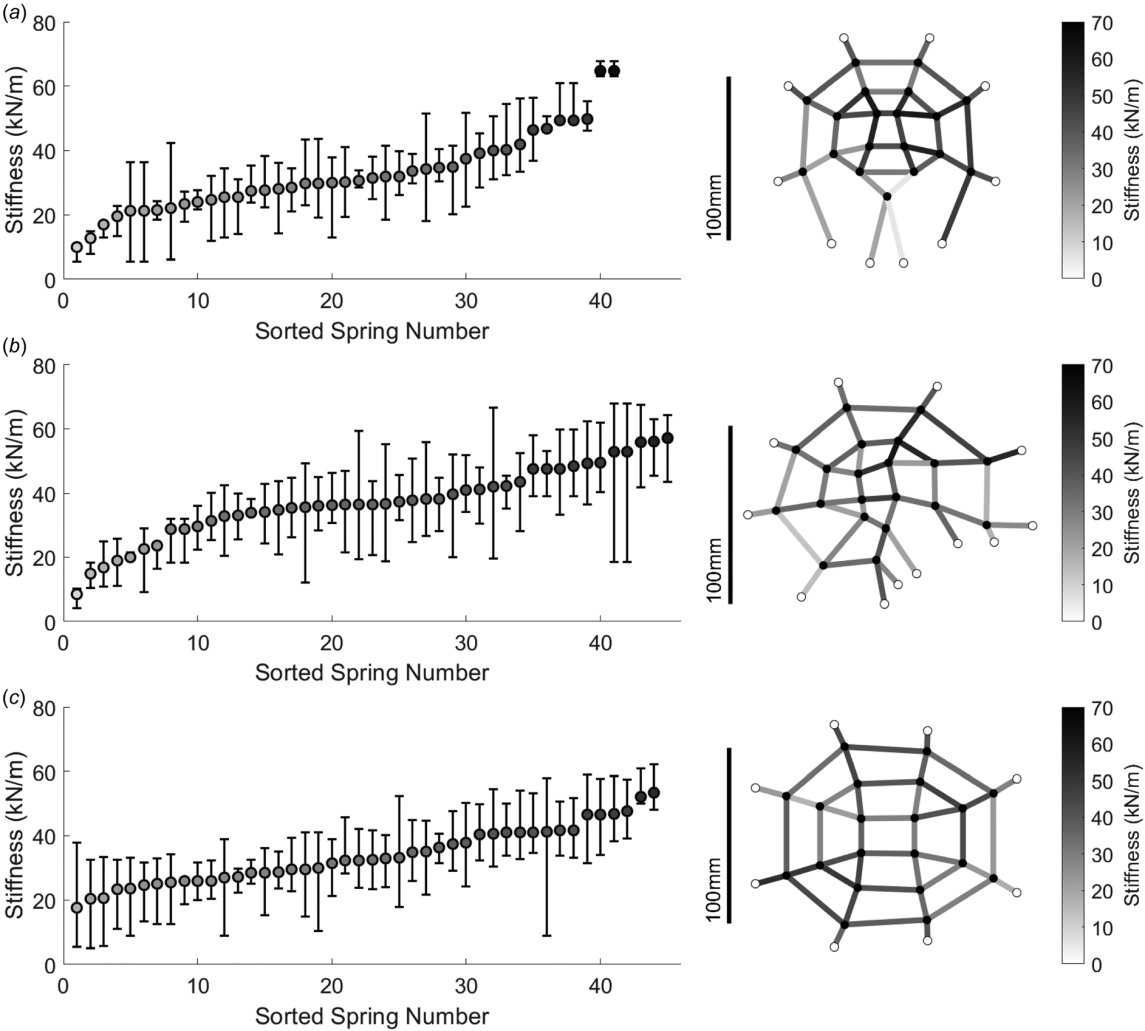
Optimized values of spring stiffness in multibody models. Spring stiffness values for the (*a*) coronal, (*b*) sagittal, and (*c*) axial planes. Springs existing in multiple subdomains were assigned the mean stiffness from subdomain optimization. Springs were color-coded based on spring stiffness and positioned between nodes as displayed in the diagram.

Minimizing the differences in the displacements of comparative nodes between the MBM and FEM led to optimized stiffness values for the springs used in each of the planar models (Fig. 3). The optimized stiffness values spanned the range of possible stiffness values for each planar model (Figs. 3(*a*)–3(*c*)). We observed no noticeable differences in the range assigned for any of the planar models, suggesting that the range chosen was sufficient to find optimal values.

**Fig. 4:**
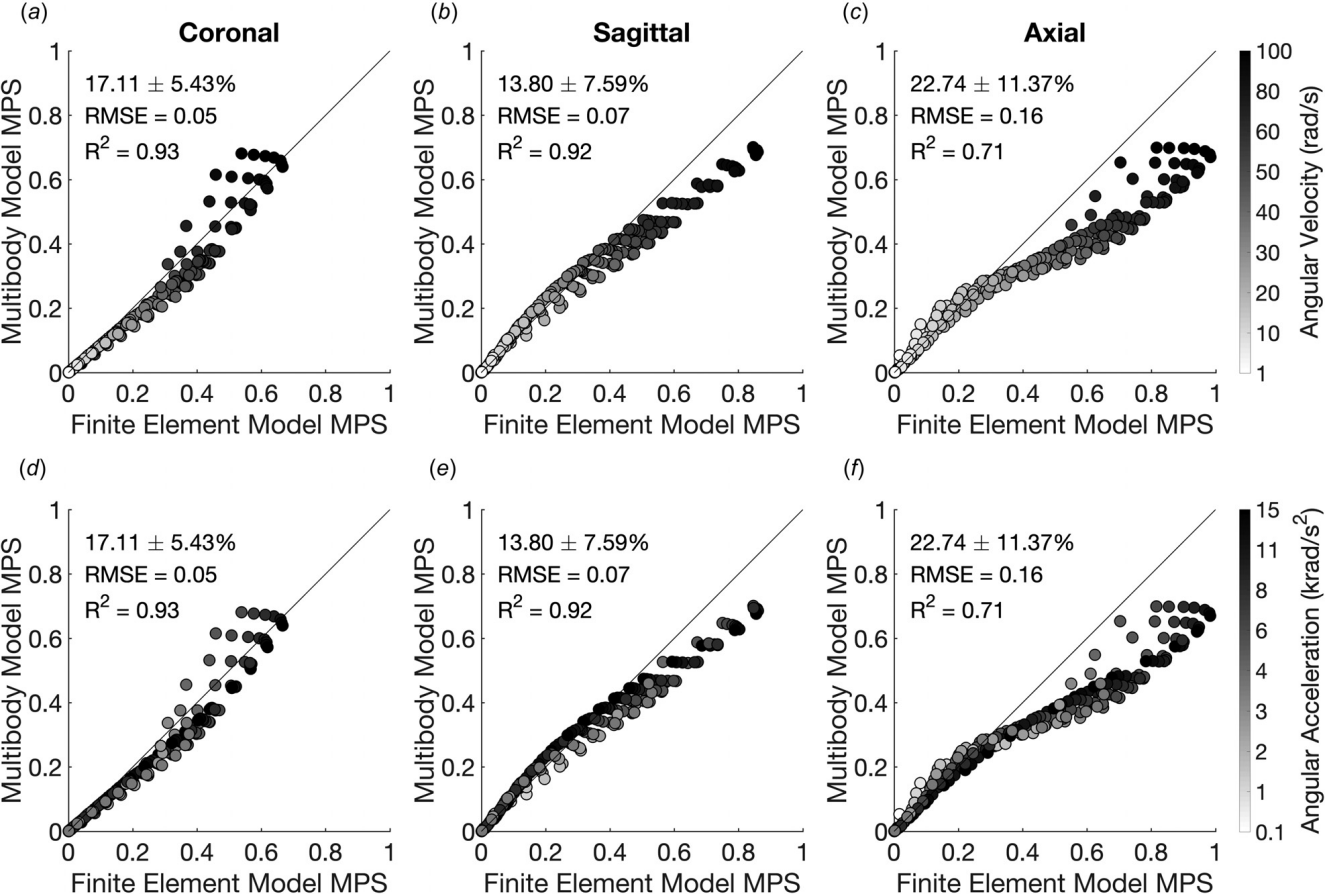
Comparison of MBM and FEM performance from sinusoidal impact traces. Cases are colored according to the peak angular velocity (*a*)–(*c*) and peak angular acceleration (*d*)–(*f*) of the impact trace. MBM closely predicted FEM MPS at low angular velocities but showed distinct differences in impact pulses with high angular velocities and low accelerations.

We next compared the predicted peak deformations between the MBMs and the FE simulations (Fig. 4). Across all three optimized MBMs, we found that the MBMs had generally good agreement with the FEM MPS values at lower-predicted MPS values. Coronal and axial plane models showed some variability in results at higher predicted MPS that was dependent on angular acceleration in high peak velocity conditions (Figs. 4(*d*) and 4(*f*)). Additionally, the sagittal and axial plane models routinely underestimated MPS values in high strain conditions (Figs. 4(*e*) and 4(*f*)). We also confirm previous results that MPS is primarily dependent on peak angular velocity and not acceleration (Fig. 4, [28]).

### Machine Learning Assists Planar Multibody Model Strain Prediction.

**Fig. 5:**
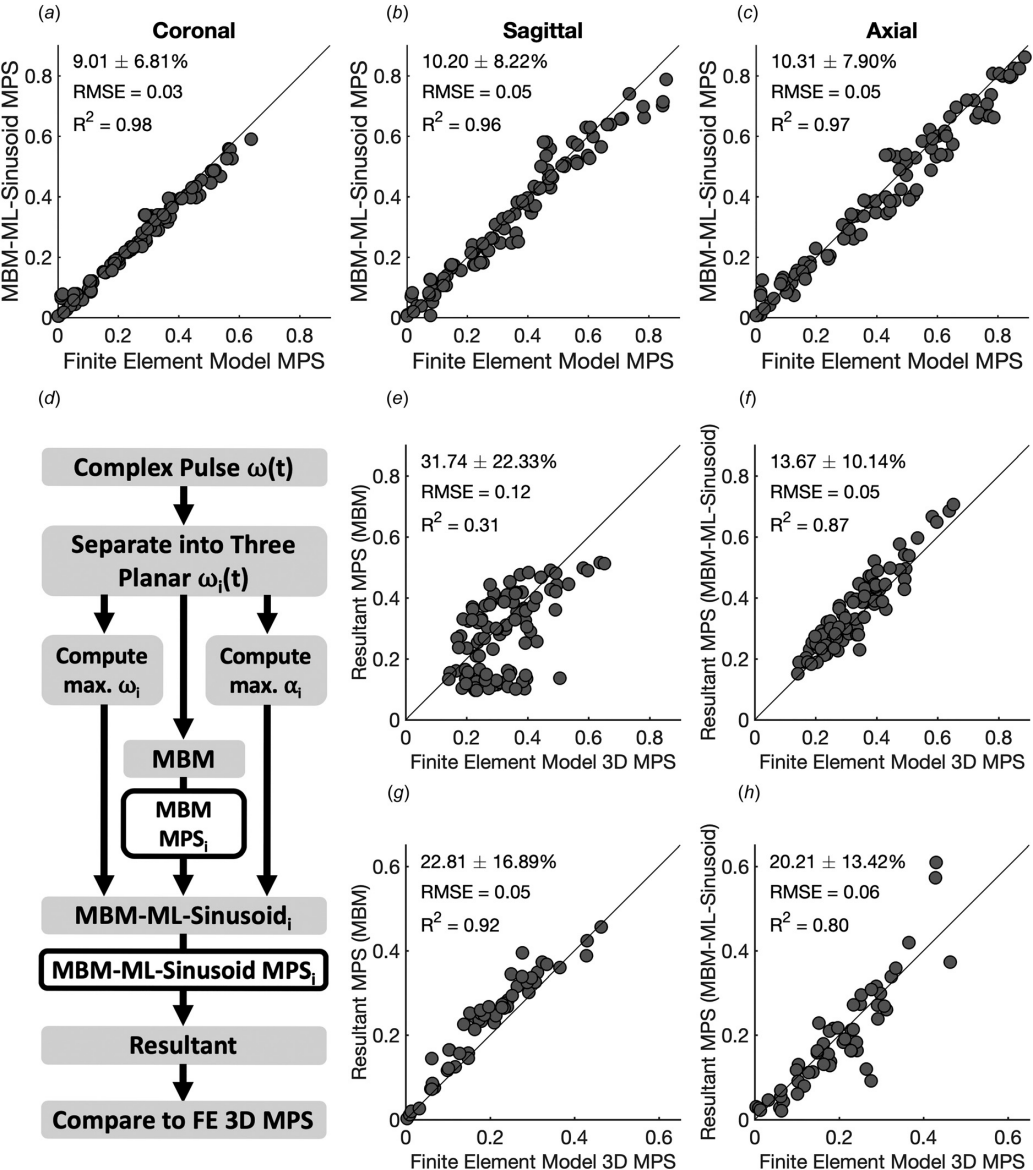
Machine learning assists MBM predictions of MPS from simple and more complex head acceleration inputs. (*a*)–(*c*) Performance of ML-assisted MBM on planar sinusoidal impact pulses. ML models were trained to predict FEM MPS using the MBM MPS and peak velocity and acceleration from the sinusoidal impact pulse (MBM-ML-sinusoid). (*d*) Flow diagram for evaluating the MBM-ML-sinusoid models with acceleration inputs from helmet impact tests, where i represents each planar direction. (*e*) MBM without the assistance of the MBM-ML-sinusoid model underestimates strain from finite element simulations of the helmet impact tests. (*f*) MBM-ML-sinusoid model improves the absolute relative error by correcting maximum principal strain estimates in each plane. MBM alone (*g*) and MBM-ML-sinusoid (*h*) models were then tested on independent human impact reconstructions.

Given potential discrepancies between the MBM and FEM, we next developed a ML model for each plane (MBM-ML-sinusoid), utilizing three features in each plane and comparing these features to the corresponding peak strain from the FEM simulations. With this approach, we observed a significant improvement in the ability to predict MPS from the 3D FEM using the MBM (Figs. 5(*a*)–5(*c*)), achieving an average absolute relative error of 9.8 ± 7.7% between the predicted and actual FEM peak deformations for each of the three planar MBMs.

Given that head acceleration exposures that may cause mTBI are rarely restricted to planar loading, we next examined the effectiveness of combining the three planar MBMs to predict the peak strain that occurred from more complex, 3D kinematics (Table 1). We simplified the 3D angular velocity input from the helmet testing data (see methods) into its three separate rotational velocity inputs, using these rotational velocity inputs for each of the three planar MBMs. We input these three kinematic loading profiles into the planar MBM-ML-sinusoid models and calculated the resultant MPS (Fig. 5(*d*)). Without utilizing the ML models, we found this approach did not yield a strong correlation between the computed peak FE strain for the complex loading profile and the estimate from the MBM (Fig. 5(*e*)). However, the resultant of the MBM-ML-sinusoid models improved our accuracy of prediction to 13.7 ± 10.1% (Fig. 5(*f*)). This model slightly overestimates full brain MPS compared with the FEM, but shows high correlation across the range of impacts tested. We additionally tested our models on human impact reconstructions and found similar performance between the pure MBM and the hybrid MBM-ML-sinusoid models (Figs. 5(*g*) and 5(*h*)). The MBM alone performed better on the human impact reconstructions (Fig. 5(*g*)) than on the helmet testing data (Fig. 5(*e*)). This can be accounted for in that the magnitudes of the impact kinematics were significantly lower (one-tailed *t*-tests, *p* < 0.001 and *p* = 0.019 for velocity and acceleration, respectively) for the human impact reconstructions. Lower kinematic magnitudes are correlated with lower strains [29], and the pure MBM performs better on smaller strains (Fig. 4).

**Table 1:**
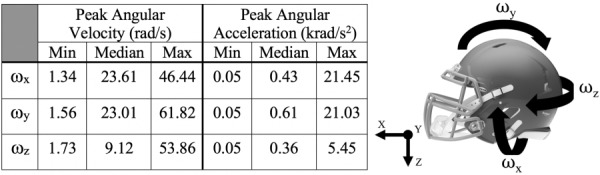
Kinematic features of helmet impact tests that show angular velocity and acceleration of helmet impact tests (*n* = 96) and a helmet with matched angular velocity rotational directions

**Fig. 6:**
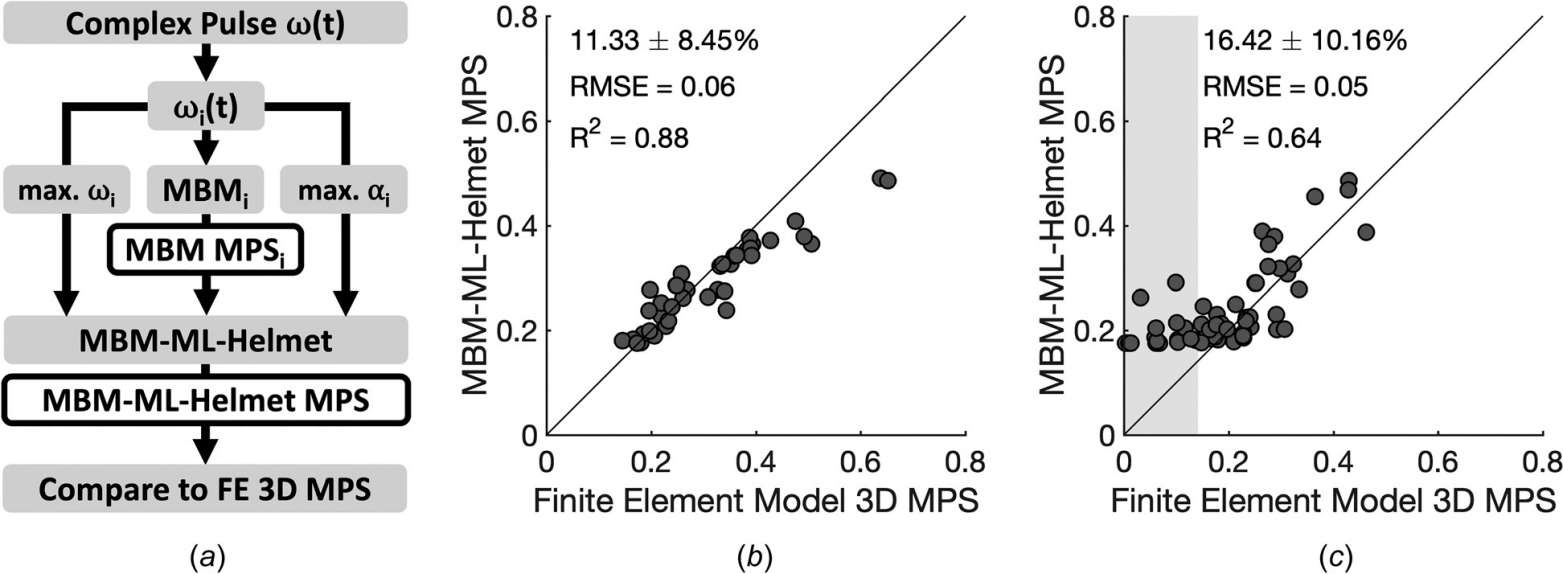
ML-assisted MBM performance after training on results from helmet impact tests. (*a*) Flow diagram for evaluating the ML model trained on helmet impact tests (MBM-ML-helmet), where *i* represents each planar direction. (*b*) MBM-ML-helmet model performance on the testing set of helmet impact acceleration inputs. (*c*) MBM-ML-helmet model performance on independent human impact reconstructions. The shaded region lies outside the lower bound MPS of the training set for the ML model, creating an MPS prediction floor. Error metrics only include points within the bounds of the training set.

While using ML to correct sources of error in the planar MBM shows promise in improving predictive capability, ML-based modeling using impact traces from helmet testing data may show further accuracy gains. We used a subset of the complex pulse inputs to train a new ML-assisted model that used the MBM estimates and peak kinematic parameters simultaneously (MBM-ML-helmet). We tested many feature sets for our MBM-ML-helmet model and found incorporating both the maximum angular velocity and acceleration of the impact traces and the MPS output from the MBM in each plane produced the best accuracy during training (Table 2). Using individual kinematic inputs (peak angular acceleration, peak angular velocity) was not as strong as combining these two features into a ML model (Table 2). However, combining the peak MPS from the MBM with either the peak angular acceleration or peak angular velocity improved the prediction accuracy relative to models using either kinematic parameter alone. We then tested the MBM-ML-helmet model on helmet testing data (Fig. 6(*a*)) and found that this model performed with an average absolute relative error of 11.3 ± 8.5% with the peak maximum principal strain computed from the FEM (Fig. 6(*b*)). As a final test, we then compared predictions from our three feature (peak multibody MPS, peak angular velocity, and peak angular acceleration) ML model using kinematic loading from reconstruction on helmet impacts in professional football [38]. Similar to the helmet testing dataset, we found that our predictions were providing comparable estimates to the peak MPS calculated from the FEM (average absolute relative error of 16.4 ± 10.2%; Fig. 6(*c*)). However, as the impact reconstruction dataset expanded below the range of the training helmet impact dataset, the predictive MBM-ML-helmet model created a minimal MPS floor of 0.18.

**Table 2:** Feature sets and performance metrics of machine learning models trained on helmet impact testing data

	Training			Validation		
Model features	RMSE	MAE	*R*^2^	RMSE	MAE	*R*^2^
MBM MPS	0.036	0.026	0.906	0.054	0.043	0.746
Peak angular velocity	0.033	0.024	0.928	0.059	0.046	0.707
Peak angular acceleration	0.038	0.029	0.897	0.066	0.052	0.617
MBM MPS, peak angular velocity	0.031	0.022	0.933	0.051	0.040	0.785
MBM MPS, peak angular acceleration	0.029	0.020	0.949	0.052	0.038	0.759
Peak angular velocity, peak angular acceleration	0.029	0.020	0.950	0.058	0.046	0.715
All	**0.028**	**0.019**	**0.953**	**0.047**	**0.035**	**0.823**

Features were drawn from each plane, e.g., the MBM MPS model included one feature from each plane. Metrics used to evaluate the training and validation of the ML models include RMSE, MAE, and correlation coefficient (*R*^2^). The model utilizing both MBM-based and all kinematics-based features (bolded) performed best.

### Machine Learning-Assisted Multibody Model Predicts Regional Strain.

**Fig. 7:**
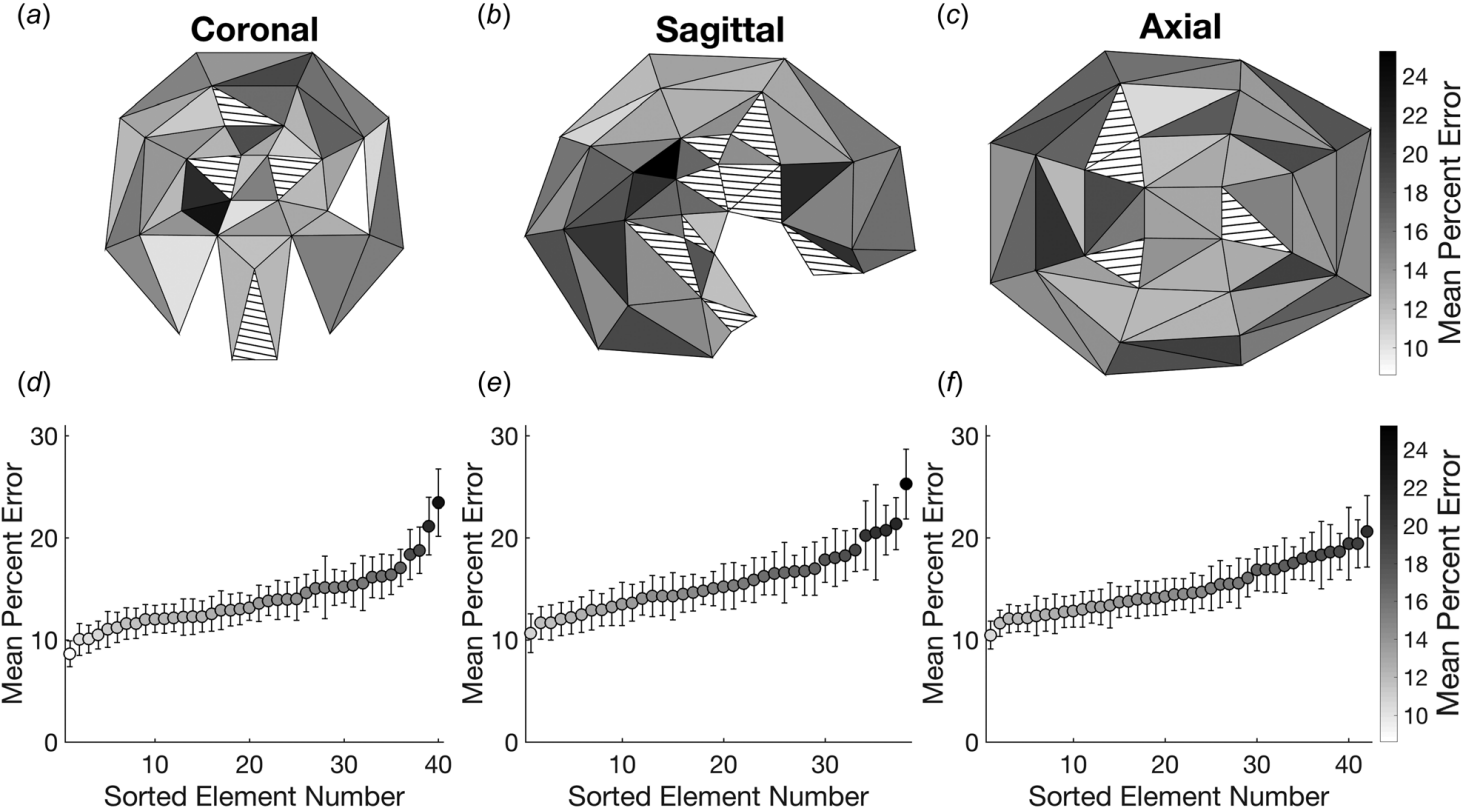
ML-assisted MBMs to predict regional MPS. Machine learning performance in the (*a*) coronal, (*b*) sagittal, and (*c*) axial planes. Triangular elements are shaded according to the mean percent relative error. Striped regions were not trained with ML models. (*d*)–(*f*) The average absolute relative error in predicted MPS for regions in each plane.

Much of the power in a detailed FEM lies in the ability to accurately represent not only the single highest value of brain deformation during an impact but strain in regional locations. As the next step in our analysis, we used a hybrid model-ML methodology to accurately predict the spatial distribution of peak principal strains throughout the brain for a given impact. Using FE simulations that computed the 3D brain response to real impact loading, we compared the strains from multibody elements spanning three adjacent nodes (triangular elements in Fig. 1(*b*)) in each of the three planar models to the equivalent FE results. For a given planar model and helmet impact kinematic inputs, we found that the correlation between the MBM elements and FEM results was reasonable. However, after training individual ML models with feature sets identical to the model used for whole-brain MPS prediction for each of the elements within a given planar MBM, the predictions improved significantly. The triangular elements in the MBM for which we created ML models had an overall absolute relative error of 14.9 ± 13.0% (Figs. 7(*a*)–7(*c*)) from the corresponding elements in the FEM, with the relative error of individual triangular elements ranging from 8.6 to 25.5% (Figs. 7(*d*)–7(*f*)).

## Discussion

In this paper, we built a MBM to predict the spatial distribution of strain across a range of impact conditions. We use simulation data from a 3D FEM of the brain subject to rapid rotation to develop and optimize this model. Further, we used ML techniques to improve the accuracy of predicting brain deformations from this MBM and show that it is possible to develop reasonably accurate estimates of brain deformation, even in response to realistic impacts, when MBMs are combined with ML algorithms.

This report builds on past studies developing rapid estimates of peak brain deformation to estimate brain injury risk. The maximum strain criterion was the first attempt to predict the relative amount of brain movement and strain from linear acceleration inputs [20]. By matching impedance characteristics derived from linear impact tests, the maximum strain criterion was used to estimate the likelihood of serious brain injury with impacts delivered across different locations on the head. More recently, the concept of precomputation emerged as a new tool to quickly estimate the peak mechanical response at different points throughout the brain for a given impact [41]. Rather than relying on computing the exact response to a specific impact condition using a 3D FEM, precomputation reverses the process and rapidly accesses a database of precomputed 3D simulations to best approximate the peak mechanical response at any point throughout the brain. Across a wide range of loading conditions, the precomputation response shows it is possible to use kinematic descriptors (peak rotational acceleration, rotational velocity) to provide reasonably good estimates of the peak brain response, as well as the response throughout different anatomic regions [41]. However, precomputation is not well suited for complex loading inputs that contain multiple impact events [42]. In contrast to these past efforts, our MBM offers the advantage of fast and reasonably accurate forward computation estimates of brain deformation for even complex rotational loading input cases, making this model particularly suitable for studying diffuse brain injuries.

Our work is most similar to a single DOF model developed recently to analyze different impact loading conditions quickly as a substitute for more intensive finite element simulations [33,34]. This type of rapid assessment tool is most relevant in crash protection and protective headgear design studies, where many different experimental values (e.g., helmet liner material, thickness; impact direction) can be tested quickly to determine which variable would most influence the brain's mechanical response. However, in generalizing the entire brain to a single mass-spring-damper, there is no ability to pinpoint possible areas of the brain that may be more likely damaged from a given impact. Our model begins to fill this gap by using a MBM to both predict the maximum strain experienced by the brain and, if desired, estimate the distribution of strain throughout this simplified model. Knowing the distribution of strain in the brain may make our model useful to predict an approximate volume of the brain exceeding specific strain threshold, matching the cumulative strain damage measure that has been used in past studies to estimate brain injury risk for a given impact exposure [28,43–45]. An alternative approach that can be used in future work is to determine whether similar accuracy for predicting strains throughout the brain could be generated by using a combination of predicted peak MPS from a single DOF model, and the relevant kinematic loading inputs along each plane.

A key technique that made our approach accurate was the inclusion of ML algorithms to better correlate the MBM prediction with the FEM simulation. Early in our analysis, we observed that discrepancies between the MBM and FEM tended to follow general trends in the kinematic loading. For example, we observed that lower angular velocity conditions showed MBM peak responses that were similar in magnitude to the FEM simulations, but this agreement soon disappeared when examining higher angular velocity conditions. The interrelationships between the kinematic inputs and MBM output responses are ideally suited for ML methods, and our significant improvement in correlating MBM output and FEM simulation shows clearly the benefit of these techniques. In recent work, similar tools were used to classify head acceleration exposure data collected with mouthguard sensors [46]. The goal of this past study was to use part of the data to train or “learn” the features that would successfully separate nonimpact and impact events, and to determine how accurate this algorithm was in classifying a separate set of data that included both nonimpact and impact events. Using one measure (peak head acceleration) in this past study poorly discriminated between nonimpact and impact events [46], much like how our MBM model prediction did not consistently track with FEM predictions. The ML methods were particularly useful for exploring a feature of the MBM that is not computed from the simpler single DOF model [33]—the distribution of maximum principal strains throughout the brain. By considering both the kinematic loading features and MBM prediction, we significantly reduced the prediction error and, on average, produced a model that differed by only 10% from the FE predictions.

Our model formulation has five primary limitations. First, we assumed that the mechanical response from a complex, 3D head rotation could be approximated by computing the peak strains from each of three orthogonal acceleration planes individually, and then recombining these into a resultant MPS [33]. Not including the potential mechanical interactions across the three planes of rotational loading is an acknowledged weakness and can be addressed in the future by developing a 3D MBM. However, developing the 3D MBM would start to increase the complexity and computational cost, e.g., roughly maintaining the same element resolution that we used in the two-dimensional models would lead to a 3D MBM of >5000 elements with a significantly longer solution time. Given the improvement in prediction offered by incorporating ML in simpler models, we explored the more computationally efficient path first. A second feature of our model was that we carefully applied our rotational loading about the center of mass for each planar model. Due to the formulation of the model, the MBM will predict significantly linear movement of nodes if a linear acceleration is applied. Such linear motion would conflict with a past work showing little to no brain motion during pure linear acceleration [15,47–50]. By using a rotation of the model about its center of mass, these effects were minimized. A third limitation is that we did not explicitly model the nonlinear properties of human brain tissue, and therefore may introduce some uncertainty in our estimates of the brain response to complex loading profiles. Fourth, our ML models are only effective when used to evaluate data that are similar to the data on which they were trained, which is a limitation inherent to ML models. Finally, our correlation of the MBM to FE simulations means that we are indirectly limited by the accuracy of the FEM. Although current FEM use much higher resolution now than models from a decade ago, the predicted deformations are influenced by a number of factors that include the choice of brain material properties, the interface between the brain and skull, and the physical size of the model [34]. We expect that our MBM, in combination with ML, is sufficiently general to evolve with new features of future FEM and therefore will continue to provide a rapid assessment tool for the community.

We recognize that this work is dependent on examining as many different impact conditions as possible to both capture the possible exposures that would lead to injury, as well as minimize the potential predictive errors that occur when using ML approaches on small datasets. This potential source of error was minimized, but not eliminated, when we divided the data into a training set and a test set, and further minimized by using cross-validation techniques to optimize the prediction from the machine learning algorithms. For human-based FEMs, we expect that our efforts to predict MPS in realistic impacts would improve with the addition of more reconstruction cases, and these are under continual development in the field [47,51–53]. Despite the drawback of using a limited number of simulations to develop our models, we are encouraged by the results from our current efforts.

In a larger scope, we expect the continued improvement of this model will offer an opportunity to advance helmet designs and player safety simultaneously. As a modeling tool, this will give helmet designers an added ability to estimate the possible benefit of any new design feature quickly during prototype testing. Although there are new computational models that can be used to evaluate new helmet designs in silico [54], these models may have difficulty capturing all of the new features in materials and shell structure that could be examined directly with helmet prototypes. Likewise, the higher resolution of the MBM may allow designers to focus on specific regions of the brain that are commonly damaged in concussive brain injury [6] when considering new helmet designs. For player safety, this tool can help better interpret head acceleration exposures measured with helmet, earpiece, or mouthguard-based accelerometer systems [55–58]. Current algorithms to predict injury risk are not based on any estimate of the brain mechanical response and suffer from low specificity [58]. Although the accuracy of these head exposure technologies may explain some of the low specificity [55,59,60], one additional factor is the inability to consider the effect of the exposure on the brain itself. Our tool would fill this need and possibly improve the ability to better separate safe from unsafe impacts.
